# Comparative mapping of the 22q11.2 deletion region and the potential of simple model organisms

**DOI:** 10.1186/s11689-015-9113-x

**Published:** 2015-07-01

**Authors:** Alina Guna, Nancy J. Butcher, Anne S. Bassett

**Affiliations:** Clinical Genetics Research Program and Campbell Family Mental Health Research Institute, Centre for Addiction and Mental Health, Toronto, ON Canada; Institute of Medical Science, University of Toronto, Toronto, ON Canada; Dalglish Family Hearts and Minds Clinic for Adults with 22q11.2 Deletion Syndrome, Division of Cardiology, Department of Medicine, Department of Psychiatry, and Toronto General Research Institute, University Health Network, Toronto, ON Canada; Department of Psychiatry, University of Toronto, Toronto, ON Canada; Centre for Addiction and Mental Health, 33 Russell Street, Room 1100, M5S 2S1 Toronto, ON Canada

**Keywords:** DiGeorge syndrome, Velocardiofacial syndrome, Animal models, Homology, Homolog, *DGCR8*, *PRODH*, *SLC25A1*, *TBX1*

## Abstract

**Background:**

22q11.2 deletion syndrome (22q11.2DS) is the most common micro-deletion syndrome. The associated 22q11.2 deletion conveys the strongest known molecular risk for schizophrenia. Neurodevelopmental phenotypes, including intellectual disability, are also prominent though variable in severity. Other developmental features include congenital cardiac and craniofacial anomalies. Whereas existing mouse models have been helpful in determining the role of some genes overlapped by the hemizygous 22q11.2 deletion in phenotypic expression, much remains unknown. Simple model organisms remain largely unexploited in exploring these genotype-phenotype relationships.

**Methods:**

We first developed a comprehensive map of the human 22q11.2 deletion region, delineating gene content, and brain expression. To identify putative orthologs, standard methods were used to interrogate the proteomes of the zebrafish (*D. rerio*), fruit fly (*D. melanogaster*), and worm (*C. elegans*), in addition to the mouse. Spatial locations of conserved homologues were mapped to examine syntenic relationships. We systematically cataloged available knockout and knockdown models of all conserved genes across these organisms, including a comprehensive review of associated phenotypes.

**Results:**

There are 90 genes overlapped by the typical 2.5 Mb deletion 22q11.2 region. Of the 46 protein-coding genes, 41 (89.1 %) have documented expression in the human brain. Identified homologues in the zebrafish (*n* = 37, 80.4 %) were comparable to those in the mouse (*n* = 40, 86.9 %) and included some conserved gene cluster structures. There were 22 (47.8 %) putative homologues in the fruit fly and 17 (37.0 %) in the worm involving multiple chromosomes. Individual gene knockdown mutants were available for the simple model organisms, but not for mouse. Although phenotypic data were relatively limited for knockout and knockdown models of the 17 genes conserved across all species, there was some evidence for roles in neurodevelopmental phenotypes, including four of the six mitochondrial genes in the 22q11.2 deletion region.

**Conclusions:**

Simple model organisms represent a powerful but underutilized means of investigating the molecular mechanisms underlying the elevated risk for neurodevelopmental disorders in 22q11.2DS. This comparative multi-species study provides novel resources and support for the potential utility of non-mouse models in expression studies and high-throughput drug screening. The approach has implications for other recurrent copy number variations associated with neurodevelopmental phenotypes.

**Electronic supplementary material:**

The online version of this article (doi:10.1186/s11689-015-9113-x) contains supplementary material, which is available to authorized users.

## Background

22q11.2 deletion syndrome (22q11.2DS, MIM #188400/#192430) is the most common micro-deletion syndrome in humans with an estimated prevalence of at least 1 in 4000 live births [1, 2]. Formerly known as velocardiofacial or DiGeorge syndrome, this multi-system condition is associated with a broad range of developmental features including congenital cardiac and palatal anomalies, intellectual disabilities, hypoparathyroidism, and subtle facial dysmorphism [2–4]. Developmental delay and later onset disorders affecting the nervous system are particularly common [5]. These include attention deficit hyperactivity disorder [6, 7], anxiety disorders [8, 9], autism [10, 11], epilepsy, schizophrenia [2, 12], and early-onset Parkinson’s disease [13, 14]. The phenotypic manifestations of the syndrome are thought to be related at least in part to reduced gene dosage in the 22q11.2 deletion region that in turn interferes with normal protein functioning [15].

The typical associated ~2.5 Mb 22q11.2 deletion is present in >85 % of individuals with 22q11.2DS [16, 17], while a smaller proximal nested ~1.5 Mb deletion occurs in ~10 % of cases [18, 19]. The associated 22q11.2 deletions are mediated by segmental duplications, or low-copy repeats (LCRs) that confer susceptibility of the region to copy number variation through non-allelic homologous recombination [20, 21]. The penetrance and variable expressivity of major associated phenotypes appear to be largely independent of deletion size [22, 23]. The few mRNA sequencing and protein expression studies of individuals with 22q11.2DS published to date [24–33] illustrate the complexity of linking specific genes to the phenotypes associated with this disorder. Much remains to be known about the individual and collective roles of 22q11.2 deletion region genes in modulating associated phenotypes. Model animals will undoubtedly play an essential role in this discovery process.

Mouse models have already been proven useful for characterizing the molecular function of 22q11.2 genes and establishing a link between certain genes and 22q11.2DS associated phenotypes [34]. The syntenic region on mouse chromosome 16 has a high degree of gene conservation to the human 22q11.2 region. Current engineered mouse models include deletions of large portions of the syntenic region and mutations of individual genes [34, 35]. However, simple model organisms could also prove to be powerful tools for investigating genomic disorders such as 22q11.2DS. Their ease of genetic manipulation, amenability to high-throughput behavioural screening, and short generation times make simple organisms attractive potential resources. The potential for simple model organisms to reveal the genetic mechanisms underlying 22q11.2DS phenotypes remains essentially unexamined however.

As an initial step in determining the utility of simple model organisms in the study of 22q11.2DS, we generated an updated, comprehensive 22q11.2DS human gene map and investigated the evolutionary conservation status of genes within the 22q11.2 region in three common model organisms: the zebrafish, *Danio rerio* (*D. rerio*), the fruit fly, *Drosophila melanogaster* (*D. melanogaster*), and the worm, *Caenorhabditis elegans (C. elegans*). We included the otherwise well-reviewed mouse models [34, 35] for comparison. We then conducted a comprehensive review of gene function and phenotypic alterations related to 22q11.2 gene homologue disruptions and developed a novel comprehensive resource of available knockout and knockdown models. The results may help to accelerate the identification of novel genotype-phenotype correlations in 22q11.2DS and inform pathogenesis of, and drug development for this disorder and its commonly associated features.

## Methods

### Human 22q11.2 region characterization

**Fig. 1:**
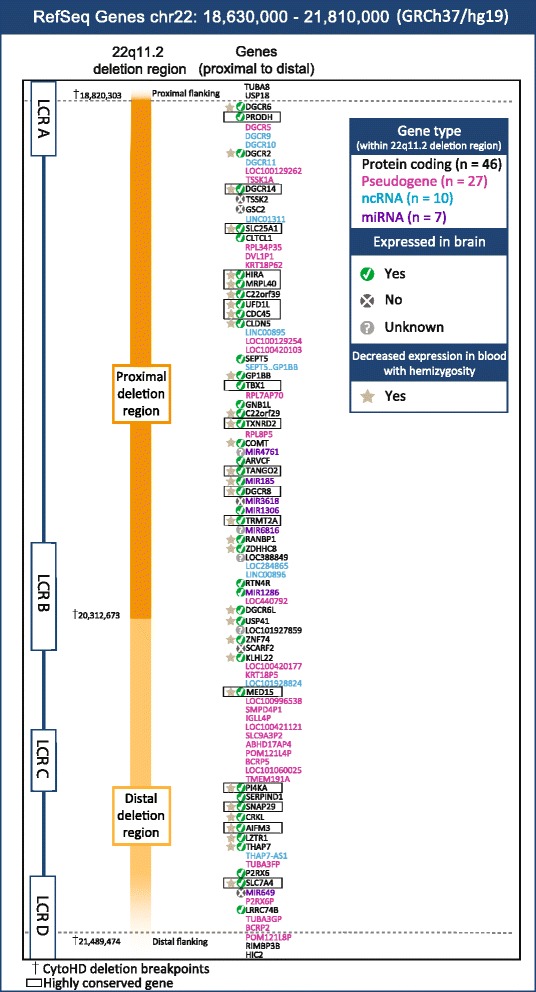
Genetic landscape of the human 22q11.2 region. The typical ~2.5-Mb 22q11.2DS deletion spans 90 RefSeq genes (see text for details). Region breakpoints are mediated by four chromosome specific low-copy repeats (LCRA-D; approximate locations shown). Gene expression, indicated by a *green circled check mark*, was established using The Human Brain Transcriptome. Data for decreased expression with hemizygosity were collated from experimentally demonstrated [24, 28–33] reductions in gene expression in blood cells from patients with 22q11.2DS. Gene names within a *rectangle* denote the 17 genes conserved across the mouse, zebrafish, fruit fly, and worm

The human 22q11.2DS deletion region, genetic content, and order were mapped from NCBI Gene *Homo sapiens* Annotation Release 105 using Affymetrix CytoScan HD (Santa Clara, CA, USA) array mean breakpoints (chr22:18,820, 303–21, 489,474) ascertained from 16 patients with confirmed 22q11.2 deletions (Fig. 1). Fourteen of the 16 patients had deletions covering most of the 22q11.2 region (~2.5 Mb) while two had smaller, nested proximal deletions. The same region was obtained with a larger, previously described patient population (*n* = 99) using Affymetrix Human SNP 6.0 breakpoints [3, 36]. All patients provided consent and the study was approved by local research ethics boards [3]. We accessed the Database of Genomic Variants to establish the corresponding locations of major UCSC segmental duplications across the deletion region (http://dgv.tcag.ca/dgv/app/home; accessed 1 December 2014). Build GRCh37 gene coordinates were used to ensure congruency across all databases used in this study. We omitted the few genes that move outside the 22q11.2 deletion region in build GRCh38 (i.e., the segment flanked by protein-coding genes *TMEM191B*…*RIMBP3*).

To identify changes in gene expression conferred by 22q11.2 hemizygosity, a systematic literature review (current to 1 December 2014) was conducted using PubMed to identify experimentally validated changes in 22q11.2 region gene expression (mRNA) in patients with 22q11.2DS. The following search terms were used: “22q11.2 deletion syndrome”, “22q11.2DS”, “DiGeorge syndrome”, “velo-cardio-facial syndrome”, “velocardiofacial syndrome”, “VCFS”, “CATCH22”, and “Shprintzen syndrome”, in conjunction with the name of each gene (and all associated gene aliases from GenBank). The Human Brain Transcriptome (http://hbatlas.org/) was used to identify genes expressed in the brain (mRNA signal ≥6) across the lifespan in any brain region [37]. For genes (*n* = 10) not found in the Human Brain Transcriptome, UniProtKB (http://www.uniprot.org/uniprot/) and associated gene expression databases (ArrayExpress, Bgee and CleanEx) were consulted.

### Gene conservation and function in model species

To identify putative orthologs of human 22q11.2 region protein-coding genes in the zebrafish (*D. rerio*), fruit fly (*D. melanogaster*), worm (*C. elegans*), and mouse (*M. musculus*), we employed the reciprocal best hits method, i.e., the protein products of genes in two different genomes represent the best hit in the opposite genome, using protein Basic Local Alignment Search Tool (blastp) analysis with the UniProtKB database (http://www.uniprot.org/uniprot/) including both Swiss-Prot and TrEMBL entries (accessed 1 December 2014). We ran blastp using each of the 46 22q11.2 deletion region protein-coding genes as a query against all proteins annotated in each genome of interest, using default settings and a maximum *E-*value threshold of 1 × 10^−6^ [38, 39]. We also required coverage of at least 50 % of any of the protein sequences in the alignments. In instances of multiple protein isoforms due to alternative splicing, the “canonical” sequence, as identified by UniProtKB, was selected for blastp analysis. To find orthologs as reciprocal best hits, we sorted blastp hits from the highest to the lowest bit score. Using this sorting method, the first hit was therefore the best hit. If the next hit had the very same score, there would be more than one hit (the method can therefore produce multiple orthologs). The same procedure was performed in the opposite direction. In the zebrafish, an organism that has undergone genome-wide duplication, we included multiple hits if the scores of putative homologues were very similar, and both hits were consistently identified across multiple databases (e.g., RefSeq). NCBI Entrez Gene was then used to individually search all putative orthologs to establish organism-specific gene location (Additional file 1). The conservation status of the seven 22q11.2 region miRNAs identified was examined using miRBase21 (accessed in December 2014) [40]. Human non-coding genes (*n* = 10) including one read-through transcript, and pseudogenes (*n* = 27) in the 22q11.2 deletion region were not investigated further.

To identify available knockout and knockdown models of the identified 22q11.2 region homologues and collate their phenotypic manifestations, we conducted a systematic search (accessed 1 December 2014) of species-specific databases including: WormBase (http://www.wormbase.org/), FlyBase (http://flybase.org/), ZFin (http://zfin.org/), and MGI (http://www.informatics.jax.org/) databases for *C. elegans*, *D. melanogaster*, *D. rerio*, and *M. musculus*, respectively. A secondary PubMed literature review confirmed that all studies examining orthologs in our model organisms of interest were included. Knockouts (homozygote) were defined as mutant models that did not produce a functional protein product due to a premature stop codon, a disruptive insertion, or full excision of a gene. For all model organisms discussed, we note only the availability of homozygous knockouts; these are more difficult to generate and are required for heterozygous knockout animals (the result of a cross of a homozygous knockout with a wild-type strain). We have however provided the known phenotypes of heterozygous knockout models for genes conserved across all examined organisms. Knockdown models were defined as those with reduced gene expression induced by any technology that interfered with the translation of a gene after it had been transcribed. The 17 genes conserved across model organisms were examined to document the availability of phenotypic information of mutant models. Single gene mutations have been reported in humans for 22q11.2DS genes, however these were outside the scope of this study.

## Results

### Characterization of the human 22q11.2 deletion region

The typical human 22q11.2 deletion overlapped 90 genes, and the smaller proximal ~1.5 Mb deletion encompassed 55 of these genes (Fig. 1). Just over half (*n* = 46, 51.1 %) of these 90 genes are protein-coding and of these, most (*n* = 41, 89.1 %) are expressed in the human brain. For the proximal nested deletion, there were 30 protein-coding genes, 27 (90.0 %) of which are expressed in the brain. We identified seven studies documenting gene expression in 22q11.2DS patients. These collectively demonstrated decreased expression of 32 (69.6 %) 22q11.2 region protein-coding genes [24, 28–33] in blood cells from patients with 22q11.2DS relative to non-deleted controls (Fig. 1). Three genes (*TBX1*, *RTN4R*, and *P2RX6P*) are not expressed in blood, [24] and the remaining 11 (23.9 %) remain to be studied in this context.

In the typical 22q11.2 region, there were also 27 pseudogenes, one read-through transcript (classified as a non-coding RNA) (*SEPT5…GP1BB*), nine non-coding RNA genes, and seven microRNAs (miRNAs; Fig. 1). Recent studies propose that the miRNA processing protein Pasha, encoded by *DGCR8*, which lies within the 22q11.2 deletion region, may play a role in modifying genome-wide expression of target genes that contribute to the neuropsychiatric phenotypes associated with 22q11.2DS, together with the region’s high density of miRNAs [36, 41–43]. Three of the seven miRNAs (MIR185, MIR1306, MIR1286) have been found to be expressed in the brain, while two were not (MIR3618, MIR649), [44] and the other two (MIR4761, MIR6816) have yet to be investigated.

### 22q11.2 region gene conservation in model organisms

**Fig. 2:**
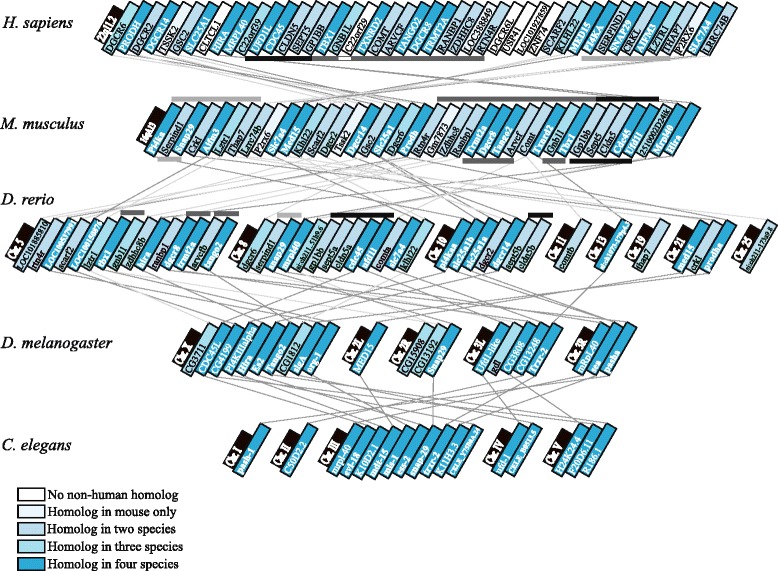
Comparative gene map of the human 22q11.2 region homologues. Protein Basic Local Alignment Search Tool (blastp) analysis on UniProtKB using the reciprocal best hits method was used to compare protein sequences to identify homologues of the 46 human protein-coding genes in the mouse (*M. musculus*), 40 conserved genes; zebrafish (*D. rerio*), 37 conserved genes; fruit fly (*D. melanogaster*), 22 conserved genes and worm (*C. elegans*), 17 conserved genes. Locations of human genes and putative homologues were mapped using NCBI Gene. *Horizontal bars* indicate clusters of two or more genes; *fine lines* otherwise join homologues between species. See text for details

The well-studied mouse syntenic region of the human proximal (1.5 Mb) deletion located on chromosome 16 (MMU 16qA13; Fig. 2) contained 27 of the 30 human protein-coding genes localized to the human proximal deletion region (Fig. 1), the exceptions being clathrin, heavy chain-like 1 (*CLTCL1*), chromosome 22 open reading frame 29 (*C22orf29*), and DiGeorge syndrome critical region-6-like (*DGCR6L*). With respect to the typical 2.5 Mb deletion, 40 of the 46 protein-coding genes are conserved in the mouse (Fig. 2).

The zebrafish also exhibited a high degree of gene conservation to the human 22q11.2DS region. In total, 37 (80.4 %) of the 46 protein-coding human homologues had putative homologues in the zebrafish (Table 1, Fig. 2). Compared to the genes contained in the human 22q11.2 region, the fruit fly had available homologues for 22 (47.8 %) protein-coding genes, and the worm had 17 (37.0 %) available homologues.

**Table 1:** Conservation status and available knockout (KO)/knockdown (KD) models of 22q11.2 deletion region protein-coding genes

Human protein- coding gene^a^	*C. elegans*			*D. melanogaster*			*D. rerio*			*M. musculus*		
	Homologue (*n* = 17)	%	Model^b^	Homologue (*n* = 22)	%	Model^b^	Homologue (*n* = 37)	%	Model^b^	Homologue (*n* = 40)	%	Model^b^
DGCR6				gdl	37	KO	dgcr6	65	−	Dgcr6	92	−
*PRODH*	CELE_B0513.5	46	KD	slgA	46	KO, KD	LOC100537991	69	−	Prodh	82	KO
							prodha	63	KO			
DGCR2							dgcr2	64	KO	Dgcr2	93	KO
*DGCR14*	ess-2	30	KO, KD	Es2	35	KD	dgcr14	67	−	Dgcr14	93	KO
TSSK2										Tssk2	92	KO
GSC2							LOC101885810	52	−	Gsc2	76	KO
*SLC25A1*	K11H3.3	65	KD	sea	67	KO, KD	slc25a1a	78	KD	Slc25a1	94	KO
							slc25a1b	84	−			
*HIRA*	K10D2.1	33	KO, KD	Hira	60	KO, KD	hira	78	KD	Hira	96	KO
*MRPL40*	mrpl-40	30	KD	mRpL40	40	KO, KD	mrpl40	50	−	Mrpl40	75	KO
C22orf39				CG15908	32	KO, KD	si:ch211..51 h9.6	49	−	2510002D24Rik	72	KO
*UFD1L*	ufd-1	41	KD	Ufd1-like	56	KO, KD	ufd1l	87	KO	Ufd1l	99	KO
*CDC45*	evl-18	29	KO, KD	CDC45L	39	KO, KD	cdc45	74	KO	Cdc45	92	KO
CLDN5							cldn5a	57	KO, KD	Cldn5	92	KO
							cldn5b	54	−			
SEPT5							sept5a	84	−	Sept5	99	KO
							sept5b	85	−			
GP1BB							gp1bb	46	−	Gp1bb	90	KO
*TBX1*	mls-1	53	KO, KD	org-1	58	KO, KD	tbx1	72	KO, KD	Tbx1	91	KO
GNB1L				CG13192	30	KO, KD	gnb1l	53	−	Gnb1l	82	KO
*TXNRD2*	trxr-2	49	KO, KD	Trxr-2	55	KD	si:ch1073-179p4.3	71	−	Txnrd2	86	KO
COMT							comta	53	−	Comt	80	KO
							comtb	54	−			
ARVCF							arvcfb	64	KO	Arvcf	92	KO
*TANGO2*	R186.1	29	KD	Tango2	30	KO, KD	tango2	64	−	Tango2	88	−
*DGCR8*	pash-1	25	KO, KD	pasha	37	KO, KD	dgcr8	72	KO	Dgcr8	95	KO
*TRMT2A*	H24K24.4	27	KD	CG3808	37	KO, KD	trmt2a	56	KO	Trmt2a	83	KO
RANBP1							ranbp1	73	KO	Ranbp1	93	KO
ZDHHC8							zdhhc8b	57	KO	Zdhhc8	92	KO
LOC388849										Gm7873	72	−
RTN4R							rtn4r	45	KD	Rtn4r	89	KO
SCARF2							scarf2	52	−	Scarf2	82	KO
KLHL22				CG1812	30	KO, KD	klhl22	60	−	Klhl22	95	−
*MED15*	mdt-15^c^	26	KO, KD	MED15	32	KO, KD	med15^b^	60	KD	Med15	90	−
*PI4KA*	CELE_Y75B8A.24	38	KO, KD	PI4KIIIalpha	45	KO, KD	pi4kaa	84	KD	Pi4ka	98	KO
SERPIND1							serpind1	54	−	Serpind1	82	KO
*SNAP29*	snap-29	28	KO, KD	Snap29	32	KO, KD	snap29	51	KD	Snap29	83	KO
CRKL							crkl	82	KD	Crkl	97	KO
*AIFM3*	F20D6.11	34	KD	CG4199	38	KO, KD	LOC100150876	66	−	Aifm3	96	−
LZTR1				CG3711	51	KO, KD	lztr1	81	−	Lztr1	95	KO
THAP7							thap7	42	KO	Thap7	93	−
P2RX6										P2rx6	86	KO
*SLC7A4*	C50D2.2	43	KD	CG13248	43	KO, KD	slc7a4	64	KO	Slc7a4	84	−
LRRC74B							si:ch211-173a9.8	41	−	Lrrc74b	68	−

^a^Genes ordered by proximal to distal 22q11.2 locus position. Genes *CLTCL1*, *C22orf29*, *DGCR6L*, *USP41*, *LOC101927859*, and *ZNF74* have no putative homologues, and are therefore not shown

^b^Knockout indicates a homozygous knockout model (heterozygous knockout models can be generated by crossing a homozygous knockout with a wild-type strain)

^c^Included due to confirmed functional homology [98], although did not meet minimal protein sequence coverage requirement at 50 %

Italicized genes have putative homologues across all four model organisms (*n* = 17). *Blank cells* indicate no identified homologue; − indicates no knockout (KO) or knockdown (KD) model available. *Percent* (%) indicates sequence similarity to human protein. Homologues were identified using blastp using the UniProtKB database. KO and KD models were identified from WormBase (http://www.wormbase.org/), FlyBase (http://flybase.org/), ZFin (http://zfin.org/), and MGI (http://www.informatics.jax.org/) for *C. elegans*, *D. melanogaster*, *D. rerio*, and *M. musculus*, respectively. Data are current to 2 December 2014

Seventeen genes were conserved across all three of the simple model species (Table 1) and in mice. By human position, these are as follows: *PRODH*, *DGCR14*, *SLC25A1*, *HIRA*, *MRPL40*, *UFD1L*, *CDC45*, *TBX1*, *TXNRD2*, *TANGO2*, *DGCR8*, *TRMT2A*, *MED15*, *PI4KA*, *SNAP29*, *AIFM3*, *SLC7A4* (Fig. 2). Notably, 16 (94.1 %) of these are expressed in the human brain (Fig. 1), five of which encode mitochondrial proteins (*PRODH*, *SLC25A1*, *MRPL40*, *TXNRD2*, *TANGO2*). In the zebrafish, there were five genes (*PRODH*, *SLC25A1*, *CLDN5*, *SEPT5*, *COMT*) where multiple putative homologues were identified, likely due to gene locus duplication [45] (Table 1; Fig. 2).

Certain genes maintained positional proximity across species (Fig. 2; Additional file 1), indicating some conserved synteny [46] (e.g., with minimal shuffling of order, from human *TBX1* to *ZDHHC8*, with an intact core from *HIRA* to *GNB1L*, to mouse *Tbx1* to *Zdhhc8,* and to zebrafish *tbx1* to *tango2*; Fig. 2). 22q11.2 region gene homologues were dispersed across 8 of the 25 zebrafish chromosome pairs, with the largest clusters involving chromosomes 5 and 8. 22q11.2 region homologues are distributed across all five fruit fly chromosomes, and all five autosomal worm chromosome pairs (Fig. 2).

No miRNAs were conserved in the zebrafish, fruit fly, or worm. The two miRNA conserved in the mouse were MIR185 and MIR1306. Human non-coding genes (*n* = 9), a read-through transcript, and pseudogenes (*n* = 27) in the 22q11.2 deletion region were not investigated in the model organisms studied.

### Availability of knockout and knockdown models of 22q11.2 region homologues in model organisms

The high proportion of conserved protein-coding genes and their arrangement in the mouse has permitted the construction of contiguous multi-gene deletion models. These include several short and long deletion models, as previously reviewed [34, 35]. Of the 40 homologous genes in the mouse, 31 (77.5 %) had available homozygous knockouts (Table 1). Notably, no mouse knockdown models were identified.

In zebrafish, of the 32 protein-coding conserved genes that did not have multiple putative homologues (i.e., excluding *PRODH*, *SLC25A1*, *CLDN5*, *SEPT5*, and *COMT*), ten (31.3 %) genes had knockouts and six (18.8 %) had knockdowns; one (3.1 %; *TBX1*) had both available (Table 1). Neither knockouts nor knockdowns were available for a substantial proportion (*n* = 15, 46.9 %) of these 32 genes. In the fruit fly, of the 22 protein-coding conserved genes, the majority (*n* = 19, 86.4 %) had both knockout and knockdown models available, two had only a knockout (9.1 %), and one had only a knockdown (4.5 %). In the worm, of the 17 protein-coding conserved genes, nearly half (*n* = 8, 47.0 %) had knockdowns available, and nine (53.0 %) had both knockdowns and knockouts (Table 2).

**Table 2:** Phenotype of available knockout/knockdown models for highly conserved 22q11.2 deletion region genes (n = 17)

	Lethality (Knockout models)^a^				Knockout and knockdown phenotypes			
Gene^b^	*C. elegans*	*D. melanogaster*	*D. rerio*	*M. musculus*	*C. elegans*	*D. melanogaster*	*D. rerio*	*M. musculus*
*PRODH*	−	No (KO)	Not assessed (KO, *prodha*); − (*LOC100537991*)	No (KO)	Reduced accumulation rate of newly synthesized proteins, extended life span, increased thermo-tolerance (KD)	Hyperprolinemia, locomotor defects including indecisive movement patterns and hypoactivity (KO); Not assessed (KD)	Not assessed (KO, *prodha*); − (*LOC100537991*)	Reduced male body weight and prepulse inhibition (genetic background dependent), impaired fear conditioning, regionally altered levels of proline (hyperprolinemia), glutamate, gamma-aminobutyric acid, and aspartate in the brain (KO)
*DGCR14*	Embryonic lethality (KO); No (het-KO)	−	−	Not assessed (KO)	Higher frequency of male progeny, meiotic prophase defect (KD); Deregulated mRNA processing (KO); Normal phenotype (het-KO)	Not assessed (KD)	−	Not assessed (KO)
*SLC25A1*	−	Embryonic lethality (KO)	− (*slc25a1a, slc25a1b*)	Majority die before 12 weeks post-natal (KO)	Normal when assessed for lethality, sterility, anatomical morphology, development, and growth (KD)	Chromosomal breaks and global loss of DNA acetylation (KO)	Mitochondrial depletion, flattened head, small heart, brain, inner ear, intestine, and mandibular arch skeleton with defect severity proportional to gene suppression, neuromuscular junction defects regardless of phenotype severity (KD, *slc25a1a*); Not assessed (*slc25a1b*)	Mice examined at two weeks are small and sickly, and show generalized hypoplasia, most severely in liver and bone marrow (KO)
*HIRA*	Embryonic lethality (KO)	Embryonic lethality (KO)	−	Embryonic lethality (KO); No (het-KO)	Not assessed (KO, KD)	Enhanced transcriptional suppression through variegation with transposable element probe, offspring of null mothers crossed with wild-type males do not develop while paternal null offspring show only partial lethality implying maternal effect (KO); Not assessed (KD)	Not assessed (KD)	Disrupted gastrulation, abnormal cardiac development (e.g., heart chambers), abnormal embryonic tissue morphology, abnormal placenta, craniofacial abnormalities, failure of brain to fuse and abnormal neural plate morphology (KO); Decreased leukocyte cell count (het-KO)
*MRPL40*	−	Embryonic lethality (KO)	−	Not assessed (KO)	Slow growth, larval arrest, reduced brood size, sterile progeny (KD)	Gross neuroanatomical defects due to under-proliferation of neuroblast cells during neurogenesis (KO); Not assessed (KD)	−	Not assessed (KO)
*UFD1L*	−	No (KO)	Embryonic lethality (KO)	Not assessed (KO); No (het-KO)	Slow growth, gonad development deficits, enlarged gut granules, locomotor defect with deviations in self-propelled movement, patchy coloration, reduced life span (KD)	Stress response reduced as determined by virus infection assay (KO); Not assessed (KD)	Decreased eye size, abnormal head shape due to hypoplasia and misarranged features, necrotic central nervous system, increased thickness of mandibular arch skeleton, hypoplastic gut and liver (KO)	Not assessed (KO); Viable with no obvious heart defects (het-KO)
*CDC45*	Embryonic lethality (KO)	Lethal in the larval stage (KO)	Not assessed (KO)	Embryonic lethality (KO); No (het-KO)	Everted vulva, reduced brood size, sterility associated with no sperm development (KO); Sister chromatid segregation defective in early embryo, reduced brood size (KD)	Gross neuroanatomy defective due to reduced cellular proliferation causing small neuroblast size in the developing brain (KO); Not assessed (KD)	Not assessed (KO)	Impaired proliferation of inner cell mass after embryo implantation (KO); Normal when assessed for size, behaviour, and sterility (het-KO)
	Lethality (Knockout models)				Knockout and knockdown phenotypes			
Gene^a^	*C. elegans*	*D. melanogaster*	*D. rerio*	*M. musculus*	*C. elegans*	*D. melanogaster*	*D. rerio*	*M. musculus*
*TBX1*	No (KO)	Partial embryonic lethality (KO)	Embryonic lethality (KO)	Embryonic lethality (KO); No (het-KO)	Abnormal uterine cell fate due to transcriptional abnormalities (KO); Normal when assessed for sterility, anatomical morphology, and development (KD)	Severely malformed or absent adult muscle precursors and supportive alary heart muscles (KO); Not assessed (KD)	Severely abnormal cardiac development (e.g., absent aortic arch), severe pouch defects and abnormal facial skeletal development, abnormal inner ear morphology (KO); Severely abnormal cardiac development (e.g., absent aortic arches) and thymus (KD)	Severely abnormal cardiac development (e.g., aortic arch), abnormal inner, middle, and outer ear morphology, abnormal lymphangiogenesis, abnormal cranial base morphology (KO); Mild cardiac abnormalities (e.g., fourth aortic arch arteries) and decreased prepulse inhibition (het-KO)
*TXNRD2*	–	–	–	Embryonic lethality (KO)	Hypersensitive to protein aggregation induced paralysis (KD) but otherwise normal when assessed for morphology and development (KO, KD)	Not assessed (KD)	–	Severe anemia and growth retardation due to perturbed cardiac development and augmented apoptosis of hematopoietic cells (KO)
*TANGO2*	–	Not assessed (KO)	Not assessed (KO)	–	Not assessed (KD)	Not assessed (KO, KD)	–	–
*DGCR8*	No (KO)	Lethal before end of pupal stage (KO)	Not assessed (KO)	Embryonic lethality (KO); No (het-KO)	Accumulation of miRNA target protein, decreased lifespan (KO); Reduced miRNA processing, accumulation of target mRNA, vulva defects, enhanced locomotor deficits of *unc* mutant (uncoordinated) phenotypes (KD)	Abnormal olfactory projection and mushroom body neuron morphology and neurophysiology (KO); Not assessed (KD)	Not assessed (KO)	Reduced dendritic spine number, reduced dendritic complexity, decreased prepulse inhibition and abnormal spatial working memory (het-KO)
*TRMT2A*	No (KD)	No (KO)	Not assessed (KO)	Not assessed (KO)	Not maternally sterile but otherwise not assessed (KD)	Not sterile (KO); Not assessed (KD)	Not assessed (KO)	Not assessed (KO)
*MED15*	Reduced lifespan (KO)	Pupal lethality (KO)	–	–	Sterile, small, increased apoptosis, decreased protein expression, changes in mRNA expression, intestinal morphology, reduced lifespan, uncoordinated locomotion (KD); Hypersensitivity to toxin exposure (KO, KD)	Abnormal wing development (KO, het-KO); Abnormal wing development and shortened legs, formation of ectopic sensory organs and induced cellular apoptosis (KD)	Disruption of dorsal/ventral patterning and mesoderm development (KD)	–
*PI4KA*	Embryonic lethality (KO)	Lethal before end of larval stage (KO)	–	Embryonic lethality (KO)	Slow growth and sterility (KD)	Abnormal eye morphology, neuromuscular junction overgrowth (KO); Not assessed (KD)	Decreased eye, head, and mesenchymal cell proliferation, increased apoptosis and necrosis of brain cells (KD)	Premature death due to degeneration of mucosal cells in the stomach and intestines (KO)
*SNAP29*	Embryonic lethality (KO)	Pupal lethality (KO)	–	Pre-weaning lethality (KO)	Defects in secretion from intestinal epithelial cells (KO); Sterility associated with endomitotic oocytes and pre-mitotic maturation of the oocyte, abnormal localization of phospholipid membrane components (KD)	Not assessed (KO); Synaptic defects characterized by abnormal basal neurotransmission. Lethality observed in the pupal stage (KD)	Disrupted pigmentation, epidermal irregular spatial pattern, disorganized keratinocyte cell surface (KD)	Not assessed (KO)
*AIFM3*	–	No (KO)	–	–	Normal when assessed for lifespan and sterility, anatomical morphology, development, and growth (KD)	Not sterile (KO); Not assessed (KD)	–	–
*SLC7A4*	–	Not assessed (KO)	Not assessed (KO)	–	Normal when assessed for sterility and anatomical morphology (KD)	Not assessed (KO, KD)	Not assessed (KO)	–

^a^Knockout (KO) indicates homozygous KO model phenotype; except where indicated as a heterozygous KO (het-KO) phenotype

^b^Genes ordered by proximal to distal 22q11.2 locus position; “–” indicates no knockout (KO) or knockdown (KD) model available. Lethality for KD models is not included as this may vary based on when the gene is suppressed

### Availability of phenotypic information for conserved 22q11.2 region genes in animal models

Examination of the 17 genes conserved across species showed substantial variability in the availability and comprehensiveness of phenotypic information for homozygous and heterozygous knockouts, and knockdown models (Table 2). In the mouse, there were 13 genes with mutants available, of which nine had some form of phenotypic characterization. Zebrafish also appeared to be under-investigated; of 12 conserved genes with mutants available, phenotypic information was available for only six. Notably, a *DGCR8* knockout has not been phenotypically assessed in zebrafish. More phenotypic information was available for mutants in the fruit fly (13 of 17 genes) and worm (15 genes), possibly due to the use of forward genetic screens in these organisms [47, 48]. Only six genes were phenotypically characterized across all three of the simple model species (*SLC25A1*, *UFD1L*, *TBX1*, *MED15*, *PI4KA*, and *SNAP29*). Findings from these phenotypic studies are discussed below in the context of clinical manifestations of 22q11.2DS. We note that, as for all genetic studies, it is important to determine whether phenotypic effects are related to a specific background strain. This is an important consideration for all model animals including the mouse [49], zebrafish [50], fruit fly [51], and worm [52]. For example, *Prodh* homozygous in mice mutants were shown to be defective in prepulse inhibition [53], but this effect was dependent on genetic background [54].

## Discussion

Here, we defined a comprehensive gene map of the most common human micro-deletion syndrome, 22q11.2DS, and conducted the first systematic examination of 22q11.2 deletion region gene conservation in simple model organisms. We developed a comprehensive resource of available knockout and knockdown models of conserved 22q11.2 region homologues. Our results demonstrate that of the human 22q11.2 region protein-coding genes, a substantial proportion is conserved in simple model organisms. These mutant model organisms are amenable to extensive phenotypic characterization. This novel comparative multi-species resource can be used to provide initial insights into how simple animal models could be used to investigate the multi-system congenital and neurodevelopmental conditions associated with hemizygous 22q11.2 deletions.

### Advantages of using a non-murine animal model in the study of 22q11.2DS

The amenability of the zebrafish, fruit fly, and worm to genetic manipulation compared with the mouse could facilitate rapid and cost-effective generation of individual targeted gene and multi-gene mutations (Table 1). Such manipulations will be essential to functional studies and gene variant interpretation of 22q11.2 region genes. Moreover, the ease of genetic manipulation could rapidly improve our understanding of how the 22q11.2 deletion may interact with the rest of the genome to mediate the variable expressivity of 22q11.2DS associated phenotypes through mechanisms such as translational modifications due to the loss of one copy of *DGCR8* and 22q11.2 region miRNA genes [55]. The relatively high proportion of 22q11.2 deletion region gene knockouts available in the mouse compared with the zebrafish and the worm is notable given that the process of developing knockout mouse models is expensive and time-consuming, calculated to take on average of about 1 year at a cost of more than US $12,000 [56]. With the advent of the CRISPR/Cas9 system, the cost and speed of developing knockouts for all organisms will substantially decrease [57].

Unlike the mouse, knockdown models for 22q11.2DS homologues are available for the zebrafish, fruit fly, and worm (Table 1). Gene knockdown technologies are advantageous because they can be used to reduce gene expression in a dose-dependent manner with a high degree of specificity [58, 59], essential for examining 22q11.2DS, where dose-dependency is thought to underlie phenotypic changes [15]. All knockdowns found for 22q11.2 homologues in the zebrafish were generated using morpholinos [60]. Knockdowns in the worm and fruit fly commonly employed RNA interference (RNAi) methods. Notably, it is possible to knockdown two genes simultaneously using combinatorial RNAi in *C. elegans* and *D. melanogaster* [61, 62]. There are, as yet, no examples of this for 22q11.2 region genes, although such experiments could yield critical insights into the possibility of epistatic effects between 22q11.2 region genes that may mediate the complex expression of 22q11.2DS associated phenotypes [54]. Although RNAi technology is available in some vertebrates including the mouse, it is rarely used due to the length of RNAi generation times and the lack of simplicity compared with invertebrates [58].

### Suitability of non-murine model organisms to study 22q11.2DS

Useful and valid model organisms should demonstrate examples of convergence with the human 22q11.2DS phenotype, thus providing proof-of-principle of the utility of these organisms in characterizing gene function and disease modeling (e.g., as we observed here for *TBX1*). Cellular and phenotypic observations already made in lower animals could identify new avenues of investigation regarding the roles of particular genes in different 22q11.2DS phenotypes. The limited data available make it difficult at present to make phenotypic comparisons between species that could help to indicate conserved molecular functions of 22q11.2 genes, especially as such data were rarely collected in the context of 22q11.2DS (Table 2). However the data reveal opportunities for novel functional studies of these genes.

One example of particular relevance to neurodevelopmental processes comes from *PRODH*, which encodes a mitochondrial enzyme that metabolizes L-proline [63], an amino acid involved in modulating glutamatergic and GABA-ergic transmission [64]. A report of severe psychomotor delay in a male with a homozygous deletion [65] indicates *PRODH* may also be an important candidate gene for motor functioning. Movement abnormalities are commonly observed in individuals with 22q11.2DS, including hypotonia in infancy, delayed gross-motor milestones in childhood [66], susceptibility to antipsychotic-induced movement disorders [67], and early-onset Parkinson’s disease [13], but it remains unclear which of the 22q11.2 genes are involved in these processes. Observations in the fruit fly, however, suggest a novel role for *PRODH* in motor pathways (Table 2). Its *PRODH* homologue, *slgA*, is prominently expressed in the nervous system during embryonic development and shows proline dehydrogenase activity [63]. Fruit flies with a homozygous *PRODH* mutation demonstrate severe locomotor defects and indecisive movement patterns compared with wild-type flies in an activity chamber assay [63]. Notably, this observation in the fruit fly spurred the study of *Prodh* in locomotion in mouse models, where the effects of *Prodh* have been less clear [53, 54]. Further study on the role of *PRODH* in mediating 22q11.2DS associated motor deficits is warranted.

Gene knockout or knockdown technologies have only recently been used in non-mouse model organisms, specifically in the context of 22q11.2DS phenotypes. One of the few examples is *SLC25A1*, a mitochondrial citrate transporter important for proper mitochondrial functioning. In zebrafish, knockdown of the *SLC25A1* homologue, *slc25a1a,* during embryonic development causes mitochondrial depletion and gross morphological defects that recapitulate some features of 22q11.2DS (Table 2). Zebrafish treated with a *slc25a1a* morpholino showed a dose-dependent phenotype, with higher doses leading to more severe developmental dysmorphic abnormalities such as a flattened head and a marked reduction in the size of the entire cranial region, including the brain [68]. Additionally, fish with marked *slc25a1a* depletion had small hearts surrounded by pericardial edema. These results indicate that *slc25a1a* plays a role in cardiac as well as craniofacial and brain development, all cardinal features of 22q11.2DS. Notably, these phenotypes were rescued in treated animals when autophagy was blocked [68]. *SLC25A1* exemplifies the potential for studies using knockdown models of simpler organisms to investigate the molecular underpinning of 22q11.2DS phenotypes that may yield novel therapeutic targets.

A novel avenue of investigation based on the high degree of gene conservation of mitochondrial genes in the 22q11.2DS region is also now indicated. Mitochondrial dysfunction is implicated in the etiology of brain-based disorders associated with 22q11.2DS, including developmental delay, schizophrenia, and Parkinson’s disease [69–71], and perturbation of mitochondria and related pathways affects key cellular processes such as cell migration, apoptosis, and synapse formation [72]. Aberrant expression of mitochondrial genes, already documented in 22q11.2DS patients for five of the six (*PRODH* remains unexamined) 22q11.2 mitochondrial genes [73], all found in the human brain (Fig. 1), could mediate susceptibility to neurological conditions in individuals with 22q11.2DS. For example, *MRPL40* knockouts in the fruit fly show defects in neurogenesis that compromise neurodevelopment (Table 2), and knockdowns of *TXNRD2* in worms overexpressing human beta-amyloid peptide as a model for Alzheimer’s disease were more susceptible to muscular dysfunction and paralysis [74]. More comprehensive studies of neurodevelopment and neurodegeneration in lower organisms could shed light on the molecular function of these critical proteins.

### Individual organism suitability for studying specific 22q11.2DS phenotypes

Like the mouse [34, 35], zebrafish, fruit fly, and worm models are unable to singularly recapitulate all of the 22q11.2DS associated phenotypes. A limitation for all animal models is the challenge presented by complex disease phenotypes, such as the range of psychiatric disorders associated with 22q11.2DS. Particularly for simple organisms, limited behaviors, and the paucity of reproducible tests make it difficult to study such phenotypes. Although a considerable number of genes are conserved in the zebrafish, fruit fly, and worm, incomplete conservation limits the ability to fully investigate the roles and possible interactions between all 22q11.2 region genes. However, these issues should not discourage the further study of specific 22q11.2DS orthologs. As discussed below for each proposed model organism, orthologs in lower animals could be very useful for clarifying the roles of 22q11.2 deletion region genes in basic neurodevelopmental trajectories (e.g. development of neuronal components), as well as organ development. For example, despite the absence of a heart in the worm, mutants of *mls-1* (the ortholog of *TBX1*) indicate that *mls-1* is involved in the specification of non-striated muscle during development [75], suggesting the potential utility for these mutants in studying how *TBX1* mediates heart development in higher organisms. Discretion is essential when deciding which model organism to use to study particular 22q11.2DS phenotypes. However, organism-specific characteristics and developmental trajectories do suggest that certain 22q11.2DS phenotypes are particularly well suited for study in each of these non-mouse models.

Congenital cardiac defects, involving cardiovascular molecular development, are major manifestations of 22q11.2DS [3] where the molecular underpinnings are difficult to analyze in mammalian models, since there is rapid death without an intact cardiovascular system during embryonic development [76]. Improvements in generating conditional gene deletion models using technologies such as the Cre-loxP and Tet-On/Tet-Off system in mice offer the potential to circumvent these issues, but have not been fully developed in the context of investigating congenital heart defects [77, 78]. Recently, the zebrafish has emerged as a highly advantageous vertebrate model for studying early cardiovascular development, largely due to the ability of the zebrafish embryos to obtain oxygen in the absence of blood circulation through passive diffusion. 1This permits survival of the initial stages of embryonic development and allows investigation of even severe cardiovascular defects [79]. The additional optical transparency of zebrafish embryos, used in combination with tissue-specific expression of fluorescent proteins, permits visualization of early molecular processes [80]. In humans, *TBX1* is associated with heart defects, palatal anomalies, facial dysmorphism, and low calcium levels [81, 82] albeit each with reduced penetrance [3]. In the zebrafish, inhibiting the *TBX1* homologue leads to developmental defects of the pharyngeal arches, aortic arches, and thymus (Table 2). Abnormal cardiac morphology was visible in roughly 20 % of knockdowns, with compromised cardiac performance in nearly all injected embryos [83]. The zebrafish provides a unique opportunity to study early development and how 22q11.2 gene dosage affects these processes through the use of knockdown technologies.

The fruit fly is particularly well-suited for the study of the myriad brain-related disorders associated with 22q11.2DS [3, 6, 9, 13, 84], including intellectual disability, and other neurodevelopmental [85] and neurodegenerative disorders [86]. The benefits of using *D. melanogaster* include its short generation time along with a high reproductive rate and the availability of powerful genetic and molecular tools. Genes of interest can be readily manipulated in a time and cell-or tissue-specific manner using well-established tools such as the GAL4/UAS*-*system [86]. Together with well-characterized developmental stages, a simple and defined nervous system, and the ability to conduct large-scale behavioral and neurophysiological assays, the fruit fly has proven a valuable tool in the study of genomic and neurological disorders [87]. Similar opportunities for study are possible for 22q11.2DS, although there have been few studies to date targeted to 22q11.2DS (Table 2). Nevertheless, studies in the fruit fly have already provided some novel insights into the molecular function of 22q11.2 deletion region genes pertinent to the associated neurological conditions. For example, in the context of identifying novel intellectual disability candidate genes in a large-scale screening study, flies with a knockdown of *SNAP29* were found to have profound synaptic defects characterized by abnormal basal neurotransmission [88] (Table 2).

To better assess the role of 22q11.2DS genes in developmental processes, additional molecular information is needed, such as where and when a gene is expressed, and elucidation of its protein-protein interactions. An ideal model for these experiments is the worm. In addition to having conserved fundamental biological processes and homology with mammals, the worm is noteworthy for being highly amenable to forward and reverse genetic screens [89, 90]. One 22q11.2DS gene provides an example of the utility of using the worm system to study 22q11.2DS is *DGCR8*, a component of the “microprocessor” complex essential for genome-wide miRNA production [91] that may mediate the expression of multiple 22q11.2DS associated phenotypes in patients, including schizophrenia [41–43]. In mice, *Dgcr8* has been implicated in altering the biogenesis of genome-wide brain miRNA [92]. Indeed, miRNAs were first discovered in 1993 in genetic screens performed in the worm and were initially thought to be phenomena unique to nematode biology [93, 94]. It was later found that miRNA are widely conserved among eukaryotes as functional non-coding RNAs [95] and their role has been further studied in the mouse [96], zebrafish [97], and fruit fly [98].

*DGCR8* was first identified as a candidate gene for miRNA processing based on a genome-wide two-hybrid analysis of *D. melanogaster* where the protein product, Pasha, was shown to interact with Drosha [99, 100]. Inactivation of a temperature sensitive allele of *DGCR8* in *C. elegans* lead to the accumulation of protein products of other genes elsewhere in the genome, particularly *let-7*, and a reduction in life span [101]. Similar mechanisms could be associated with the unexplained premature mortality reported in individuals with 22q11.2 DS [102]. In another study investigating gene-gene interaction mechanisms, RNAi targeting the worm *DGCR8* homologue resulted in exacerbation of the uncoordinated motor phenotype caused either by a mutation in the human tau-FTDP-17 homologue or by *unc* (uncoordinated) mutations [103]. This is of interest in light of motor defects seen in patients with 22q11.2DS [104, 105]. Comparable interactive mechanisms, involving effects of hemizygosity of DGCR8 on expression of mRNA across the genome, may also be operating in 22q11.2DS [41–43]. Differences in mRNA expression may also be mediated by another 22q11.2 region gene, *DGCR14*, whose ortholog in *C. elegans* has been implicated in promoting proper mRNA splicing when splice sites are compromised [106]. Notably, conservation is a key means of determining relevance of a non-protein-coding sequence in humans [107].

Another as yet unexplored area in the context of model organisms and 22q11.2DS relates to the development of pharmaceutical agents to treat 22q11.2DS related phenotypes. Collectively, *D. rerio*, *D. melanogaster*, and *C. elegans* are particularly suited to screening chemical libraries for potential drug development, circumventing the high financial and time investment for *M. musculus* [108].

### Study limitations

We used a reciprocal best hits method to identify putative orthologs of human 22q11.2 genes in simple model organisms, a common and well-established method to probe orthology based on sequence similarity [39]. In this exploratory study, we did not restrict to a minimum sequence identity in order to identify all putative orthologs of the human 22q11.2 genes. Similarity in protein sequences does not necessarily translate to conserved function or patterns of gene expression across species. Further experiments are needed to assay possible conserved functional roles [109, 110] of the homologues identified here. Importantly, we found that our results were consistent with previous homology relationships described for 22q11.2DS genes (e.g., *PRODH* [111], *UFD1L* [112], *DGCR6* [113], *MED15* [114], *TSSK2* [115], and *TXNRD2* [116]). Using these methods, we identified multiple putative homologues for individual 22q11.2 deletion region genes in the zebrafish, possibly related to gene duplication [45]. In these cases, functional studies are required to examine which homologue may have a conserved function. Additionally, using the stringent criteria for the reciprocal best hits method, homology may be difficult to establish for genetically distant species. For example, query coverage was too low to identify a reciprocal best hit for the mitochondrial gene *ZDHHC8* in the fruit fly and worm. Our analyses were restricted to protein-coding genes and miRNAs. The examination of other non-coding genes will be deferred due to the limited resources available as of yet. For the 17 genes conserved across all species examined, we noted some discrepancies in mutant phenotypes across species that could suggest divergent gene functions (Table 2). However, this is likely related to the absence of studies of comparable phenotypes (e.g., the cardiovascular system investigated in one organism but not another) and the relative sparseness of available data for many genes.

## Conclusions

These results indicate that the zebrafish, fruit fly, and worm are valuable but underused model organisms in the study of the important human genetic syndrome, 22q11.2DS, associated with a 22q11.2 deletion involving 46 protein-coding genes. There is a relatively high degree of conservation of these genes, and some relevant models already exist in non-murine organisms. Despite this, few have been studied in the context of 22q11.2DS. Manipulating expression of 22q11.2 region genes in the zebrafish, fruit fly, or worm can reveal phenotypic manifestations relevant to 22q11.2DS and a greater understanding of their molecular origins in ways that are not possible in humans or readily possible in mouse models. In addition to providing a novel comprehensive comparative resource for the many developmental genes in the 22q11.2 region, we illustrate a proof-of-principle of the utility for applying a similar approach to investigating other pathogenic copy number variants associated with multi-system developmental disorders and important neurodevelopmental phenotypes.

## Additional file

Additional file 1:
**22q11.2 gene homologue locations.** The human 22q11.2DS deletion region genetic content and order was mapped from NCBI Gene *Homo sapiens* Annotation Release 105 using Affymetrix CytoScan HD (Santa Clara, CA, USA) array mean breakpoints (chr22:18,820,303–21,489,474) ascertained from 16 patients with confirmed 22q11.2 deletions. Protein Basic Local Alignment Search Tool (blastp) analysis on UniProtKB was used to identify putative homologues using the reciprocal best-hits method. NCBI was used to find gene coordinates of putative homologues. All data were recent to 2 December 2014. See Methods for additional details.
